# Potential of Cocoa Pod Husk Pectin-Based Modified Release Capsules as a Carrier for Chronodelivery of Hydrocortisone in Sprague-Dawley Rats

**DOI:** 10.1155/2018/9825363

**Published:** 2018-10-08

**Authors:** Ofosua Adi-Dako, Kwabena Ofori-Kwakye, Seth Kwabena Amponsah, Isaac Boamah, Noble Kuntworbe, Esther Eshun Oppong

**Affiliations:** ^1^Department of Pharmaceutics, Faculty of Pharmacy and Pharmaceutical Sciences, College of Health Sciences, Kwame Nkrumah University of Science and Technology (KNUST), Kumasi, Ghana; ^2^Department of Pharmaceutics and Microbiology, University of Ghana School of Pharmacy, Accra, Ghana; ^3^Department of Pharmacology and Toxicology, University of Ghana School of Pharmacy, Accra, Ghana; ^4^Department of Medical Microbiology, School of Biomedical & Allied Health Sciences, University of Ghana, Accra, Ghana; ^5^Department of Pharmaceutics, School of Pharmacy, Central University, Miotso, Ghana

## Abstract

The potential of cocoa pod husk (CPH) pectin-based modified release (MR) capsules as a carrier for chronodelivery of hydrocortisone in Sprague-Dawley rats was assessed. Extemporaneously formulated CPH pectin-based hydrocortisone (10 mg) capsules crosslinked with calcium chloride (Formulation A) or zinc (Formulation B) and a commercial immediate release hydrocortisone formulation were administered orally to Sprague-Dawley rats and the pharmacokinetic parameters were evaluated using noncompartmental analysis. Formulation A had a 2 h lag phase followed by an increase in serum drug concentration in the treated rats. Peak concentrations (C_max_) of 21.799 ± 1.993 ng/ml and 20.844 ± 2.661 ng/ml were achieved after 6 ± 0.23 h and 6 ± 0.35 h (T_max_), respectively, for capsules A and B. The immediate release formulation had a peak concentration of 15.322 ± 0.313 ng/ml within 1 ± 0.2 h. The relative bioavailability of the CPH pectin-based capsules A and B was 213% and 274%, respectively. Formulations A and B had half-lives more than three times that of the immediate release formulation. The MR capsules exhibited a higher exposure, greater bioavailability, and versatility in release of cortisol than the commercial immediate release formulation. Additionally, the MR capsules exhibited an extended drug release with overnight cortisol rise and early morning cortisol peak and hold promise in the management of adrenal insufficiency.

## 1. Introduction

Drug delivery could be optimized through the design, manufacturing methods employed, and the biopharmaceutical considerations made during formulation development [1]. The formulation of novel or optimized drug delivery platforms, intended to improve human health, has been the focus of research in recent years [2]. The symptoms of some diurnal diseases follow a rhythmic pattern and require drug delivery as per the rhythms. However, when there is constant drug release in such disease states, there is the potential for side effects as the undesired drug released tends to affect the body's metabolic system. There is therefore the need for the drug delivery patterns of such formulations to be optimized to suit unique therapeutic needs.

Cortisol is an essential metabolic hormone secreted by the adrenal cortex in a circadian fashion. The highest levels of cortisol are observed in the morning whiles the lowest levels are detected around midnight [3, 4]. A disruption of the diurnal rhythm of cortisol causes fatigue, depression, ill health, and insulin resistance. Patients suffering from adrenal insufficiency have low levels of secreted cortisol. The disease progresses in a gradual and subtle manner with fatal consequences [5, 6]. Aside adrenal insufficiency, other disease conditions that display circadian variation in their pathophysiology include hypertension, asthma, and gastric ulcer. Such disease conditions require drug delivery to mimic this rhythmic pattern [7].

Drug tolerance and side effects are associated with drugs that are released at a constant rate in diurnal disease states [8–10]. The administration of immediate release hydrocortisone (drug form of cortisol) in the treatment of adrenal insufficiency has proven to be ineffective due to inability of drug to mimic the rhythm of endogenous cortisol. To address the deficiencies associated with the use of immediate release hydrocortisone preparations in adrenal insufficiency, various modified release hydrocortisone formulations which are intended to replicate the physiological cortisol rhythm have been produced with varying degrees of success [11–14].

There is, however, the need to develop modified release hydrocortisone with optimized drug delivery to achieve enhanced and optimal blood drug concentration-time profiles [2, 15]. Preclinical studies using animal models provide critical information about the biopharmaceutical and pharmacokinetic properties of such formulations [16]. These studies serve as predictors of possible effects of these formulations in humans [17].

Cocoa pod husks (CPH), which are the empty pod shells of the cocoa (*Theobroma cacao*) fruit, have been shown to have suitable physicochemical properties as a pharmaceutical excipient [18]. An* in vitro* evaluation of the CPH pectin-based formulation as a carrier for chronodelivery of hydrocortisone demonstrated its potential use in adrenal insufficiency [14]. Findings from this earlier* in vitro* study showed that formulated CPH pectin-based systems containing hydrocortisone can achieve a burst release of the drug at circadian timing after approximately 6 hour lag phase [14]. The burst release of the drug at circadian timing was attributed to enhanced solubility and degradation of pectin in simulated colonic medium. The current study sought to design a simple formulation of a CPH pectin-based oral multiparticulate matrix system which is suitable for chronodelivery of hydrocortisone. An evaluation of the pharmacokinetic profiles of the CPH pectin-based hydrocortisone systems compared with an immediate release commercial hydrocortisone formulation was undertaken in Sprague-Dawley rats.

## 2. Materials and Methods

### 2.1. Materials

Micronized hydrocortisone powder ~50 *μ*m (Sigma Aldrich, USA), commercial 10 mg hydrocortisone tablets (Auden McKenzie, UK), calcium chloride (Fischer Scientific, UK), dicalcium phosphate (Sujian Modern Chemical, China), hydroxypropyl methylcellulose [Methocel 44779 HPMC (viscosity: 40-60 cps, 2% aqueous solution)] (Alpha Aesar, USA), microcrystalline cellulose (Blanver Farmoquimica, Brazil), maize starch (Vasa Pharmachem, India), zinc acetate (Central Drug House, India), dexamethasone injection (Lab Sanderson SA, Chile), gelatin capsule shells (Capsule Connection, USA), ether (Merck, Germany), and freeze dried CPH pectin extracted from minced fresh cocoa pod husks by hot aqueous extraction [18] were used for the study

### 2.2. Preparation of Modified Release CPH Pectin-Based Hydrocortisone Capsules

Modified release pectin-based hydrocortisone capsules containing ~10 mg hydrocortisone were extemporaneously prepared (Table 1). The stipulated amounts of the powders, except CPH pectin, were weighed and mixed by geometric dilution. CPH pectin was dispersed in sufficient amount of hot water to form a viscous dispersion and added gradually to the mixed powders to form a wet mass. The wet powder mass was passed through a #20 sieve and the granules dried at 40°C for 1.5 h in an oven. The moisture content of the granules was determined by weighing 1 g of the granules into each of three Petri dishes and dried in a hot air oven at 105°C to constant weight. The moisture content was calculated as the ratio of the weight of moisture loss to weight of sample expressed as a percentage [18]. The particle size and surface characteristics of the granules were evaluated with a Jeol Scanning Electron Microscope (JSM-6390LV, Japan) by placing samples on double-sided carbon adhesive tapes on specimen stubs and coated with platinum in the autofine coater. The samples were then scanned and viewed.

The capsules containing ~200 mg granules each were prepared manually by the traditional punch method. The granules were poured onto a powder weighing paper and the base of capsule size “0” was held vertically and the open end repeatedly pushed or punched into the powder until the capsule was filled. The cap was replaced to close the capsule. Each filled capsule was weighed using an empty capsule as a counterweight and granules were added or removed until the correct weights were obtained [19, 20].

### 2.3. Weight Analysis of Capsules

Ten capsules were individually weighed. The contents of each capsule were removed and the weights of the empty shells recorded separately. The difference between both weights was calculated and the net weight of the contents was determined for each capsule [21].

### 2.4. Content Analysis of Capsules

The hydrocortisone content of the capsules was determined using a validated Reversed-Phased High-Performance Liquid Chromatography (RP-HPLC) method [14, 22], with minor modifications. Analysis was done using a JT Baker ODS C18, 5 *μ*m, 4.6 × 150 mm column; injection volume: 20 *μ*L; flow rate: 1 ml/min; wavelength of detection: 254 nm; and temperature: ambient. The contents of ten capsules of each formulation were removed and finely powdered separately. A quantity of powder equivalent to 2.5 mg hydrocortisone was accurately weighed. The powders were transferred into 10 ml of methanol in 25 ml volumetric flasks, sonicated, and made up to 25 ml volume with methanol. The solutions obtained were filtered using Whatman filter paper No. 41, and the filtrates were analyzed by RP-HPLC. The peak areas of the samples were determined and the amount of hydrocortisone extrapolated from the linear regression calibration curves.

### 2.5. In Vitro Drug Release Studies

The release of hydrocortisone from the CPH pectin-based capsules was monitored using a USP Dissolution Tester Apparatus II (Lid-8 Dissolution Tester, Vanguard Pharmaceuticals Machinery Inc., USA). The dissolution medium was 900 ml phosphate buffer pH 6.8; the paddle speed was 50 rpm; and the test temperature was 37 ± 0.5°C. At specified time intervals, 5 ml aliquots were drawn for analysis and replaced with the same volume of dissolution medium. The hydrocortisone levels were detected with a Spectroquant Pharo 300 UV Spectrophotometer (Merck, Darmstadt, Germany). Three replicate determinations were made for each capsule formulation.

### 2.6. Experimental Animals

Eighteen male Sprague-Dawley rats (200-250 g) were obtained from the Animal House of the Centre for Plant Medicine Research (CPMR) Mampong-Akuapem, Ghana. The animals were housed in stainless steel wire mesh cages, and supplied with water and fed with chow (Agricare Co Ltd., Accra)* ad libitum*. Each rat was made to occupy a minimum space of 2 cubic feet (61 cm × 31 cm × 31 cm) with soft wood shavings as bedding for their comfort. The animals were acclimatized for 7 days and maintained under standard laboratory conditions (temperature: 25 ± 1°C, relative humidity: 60-70%, and 12 hour light-dark cycle). The animals were randomly grouped into six rats per group (Groups 1, 2, and 3). The study was conducted in accordance with the guidelines of the Institutional Research Committee of CPMR responsible for animal care and use, as well as the National Institute of Health Guidelines for the Care and Use of Laboratory Animals [23].

### 2.7. Administration of Hydrocortisone Formulations

Endogenous cortisol of the experimental rats was suppressed with 50 *μ*g/kg of dexamethasone before the administration of the hydrocortisone formulations [24]. A commercial immediate release hydrocortisone tablet (40 mg/kg) was orally administered to Group 1 rats. The modified release CPH pectin-based capsule Formulations A and B were administered orally (40 mg/kg) to Groups 2 and 3 rats, respectively. All administrations were done at 21:00 h GMT.

### 2.8. Blood Sampling and Serum Cortisol Determination

Blood sampling of rats in Group 1 was at 30 min intervals (based on half-life of immediate release formulation) from 21:00 h to 22:30 h GMT, and at 01.30 h GMT. Blood sampling of Group 2 and Group 3 rats was at 2 h intervals (based on formulation type, delayed release) from 21:00 h to 5:00 h GMT. Parallel blood sampling approach and a sampling time span of 8 h were used to reduce stress among the rats. Stress among animals could lead to an increase in levels of endogenous cortisol, which invariably affect assay of administered cortisol (hydrocortisone). Animals were mildly anesthetized with ether vapor, and blood samples collected via cardiac puncture into BD-serum separator vacutainer tubes and immediately centrifuged to obtain serum. All serum samples were stored at 4°C and analyzed for cortisol levels within 24 h. The serum concentration of cortisol was measured using a competitive immunoassay (Rat cortisol Elisa kit, Cusabio, South Africa) with an analytical sensitivity of 0.049 ng/ml. The intra-assay and interassay precision coefficient of variation were < 8% and < 10%, respectively.

### 2.9. Data Analysis

Results were expressed as mean (with standard error) and percentages. The* in vivo* pharmacokinetic parameters of the hydrocortisone formulations in the 3 groups were estimated with noncompartmental analysis using GraphPad Prism 7.0 and Excel 2013. Peak drug concentration (C_max_) and time to achieve this (T_max_) were determined from concentration-time profile curves. The area under the drug concentration-time curve (AUC) was calculated by the linear trapezoidal rule. AUC was determined till the last sampling point and was also extrapolated to infinity (AUC_0→∞_). The elimination rate constant (Ke) was determined by linear regression of the linear terminal part of the log serum cortisol concentration-time curve. The elimination half-life (t_1/2_) was calculated from the formula; t_1/2_ = 0.693Ke^−1^. Comparison of pharmacokinetic parameters of the hydrocortisone formulations was by one-way analysis of variance (ANOVA) followed by Tukey's* post hoc* multiple comparison test. Statistical significance was set at p < 0.05.

## 3. Results

**Figure 1:**
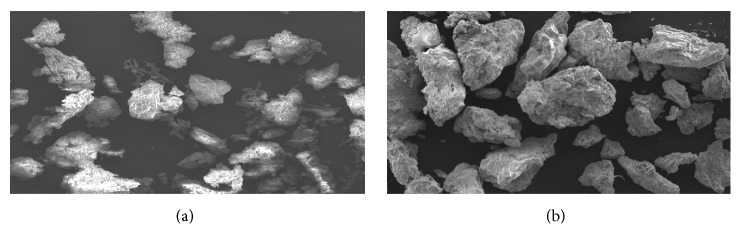
Scanning electron micrographs of (a) CPH pectin/calcium/HPMC hydrocortisone granules [Formulation A] (mag x 200) and (b) CPH pectin/zinc hydrocortisone granules [Formulation B] (mag x 50).

**Figure 2:**
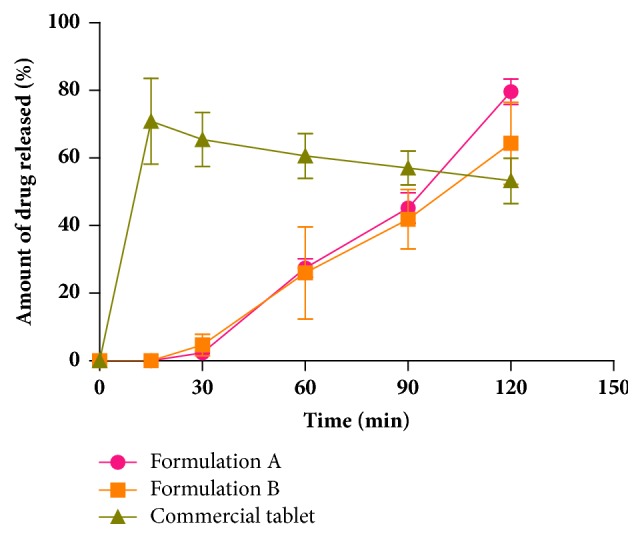
Dissolution profiles of the two CPH pectin-based hydrocortisone modified release capsule formulations and a commercial conventional 10 mg hydrocortisone tablet in phosphate buffer pH 6.8 (mean ± SEM, n = 3).

The CPH pectin-based hydrocortisone granules were irregularly shaped with rough surfaces (Figure 1). The mean particle size and moisture content of the granules were Formulation A (1.153 ± 0.19 mm, 0.02%) and Formulation B (1.858 ± 0.25 mm, 0.09%), respectively. The two modified release CPH pectin-based hydrocortisone capsule formulations prepared showed satisfactory physicochemical properties. The average weight and hydrocortisone content of Formulation A were 200.76 ± 0.45 mg and 95.99 ± 0.34%, respectively, and those of Formulation B were 200.99 ± 0.59 mg and 103.29 ± 0.25%, respectively. The capsule weight deviations were less than 7.5%.* In vitro* drug release of the modified release capsules (Formulations A and B) and the commercial immediate release hydrocortisone tablet showed biphasic release of hydrocortisone (Figure 2). Formulation A, a matrix of CPH pectin, calcium chloride, dicalcium phosphate, and HPMC containing hydrocortisone, showed negligible drug release within the first 30 minutes, after which a continuous release of hydrocortisone was observed. Approximately 28% and 79% of Formulation A was released within 1 h and 2 h, respectively. The release profile for Formulation B, a matrix of CPH pectin, zinc, and hydrocortisone, was similar to Formulation A with negligible release within the first 30 min followed by increased drug release. In all, approximately 20% and 64% of Formulation B was released within 1 h and 2 h, respectively. Even though Formulation B appeared to show a slower release of hydrocortisone than Formulation A, the difference was not significant (p> 0.05). The first phase of drug release is due to initial wetting-swelling of pectin and HPMC (Formulation A) or pectin (Formulation B), followed by diffusion of the drug through the gel matrix. The commercial hydrocortisone product exhibited accelerated drug release (71%) within the first 15 min followed by continuous decrease in release. The initial phase of drug release could be attributed to diffusion of drug molecules from the tablet in direct contact with the aqueous medium. The second phase of continuous but prolonged decrease in drug release may be due to the poor solubility of hydrocortisone in aqueous medium.

**Figure 3:**
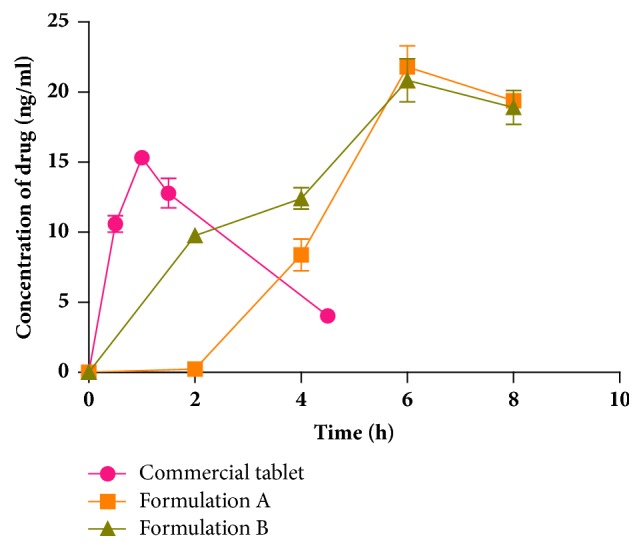
*In vivo* concentration-time profiles of the modified release CPH pectin-based hydrocortisone (10 mg) capsule formulations and a commercial conventional 10 mg hydrocortisone tablet in Sprague-Dawley rats (mean ± SEM, n = 3).

The cortisol concentration-time data (absorption profiles) obtained alternatively from 3 rats in each group (average) for Formulations A and B and the commercial conventional hydrocortisone tablet is shown in Figure 3. As a result of the abrupt end of the concentration-time curve for Formulations A and B, extrapolations were made to obtain postabsorption-elimination phases so as to be able to calculate AUC_0→∞_, t_1/2_, and Rel. F (%). As a result, no statistical comparisons are made for these 3 pharmacokinetic parameters among the 3 groups. Formulation A had a 2 h lag phase followed by an increase in serum drug concentration. The lag time observed for Formulation A could be attributed to the decreased solubility of the pectin/calcium/HPMC drug matrix in the gastric medium.

The pharmacokinetic parameters C_max_, T_max_, AUC_0→∞_, and t_1/2_ of the drug formulations are summarized in Table 2.

The administration of the oral immediate release hydrocortisone product resulted in a maximum concentration (C_max_) being obtained within 1 ± 0.2 h. With regard to the modified release Formulations A and B, the time (T_max_) to reach C_max_ was achieved after 6 ± 0.23 h and 6 ± 0.35 h, respectively. Post hoc analysis showed that the T_max_ for Formulations A and B were statistically different from the commercial immediate release formulation (p<0.001). Post hoc analysis also showed that there was a significant difference in C_max_ between Formulation A and the commercial product and between Formulation B and this immediate release product (p<0.05). However, there was no significant difference in C_max_ between Formulations A and B (p>0.05).

## 4. Discussion

The treatment of adrenal insufficiency with conventional hydrocortisone dosage forms is challenging and generally leads to suboptimal drug serum levels. Studies involving oral modified release formulations of hydrocortisone have been shown to mimic a circadian-based physiological cortisol release profile than the conventional release hydrocortisone formulations [11, 25]. The overnight rise and morning peak of cortisol is a crucial aspect of the replication of endogenous cortisol circadian rhythm when hydrocortisone is used.

In the current study, the potential of CPH pectin-based capsule system in providing an extended release of hydrocortisone during the nocturnal cortisol gradual rise and morning peak was evaluated in Sprague-Dawley rats. Two CPH pectin-based oral multiparticulate matrix capsules were formulated to achieve suitable modified release of hydrocortisone* in vitro *and* in vivo*. CPH pectin was modified to reduce its solubility in the upper gastrointestinal tract with combinations of HPMC, calcium, and zinc to achieve colon specific release of hydrocortisone and ultimately the desired release profile* in vivo* [26–28].

Content analysis of the CPH pectin-based hydrocortisone capsules yielded results in the acceptance criteria of 90-110% [29]; hence the formulations contained the requisite amount of hydrocortisone. The capsules were also within the stipulated pharmacopoeial limits of capsule weight variation. Commercial immediate release hydrocortisone preparations are routinely administered to attain acceptable therapeutic levels of cortisol in adrenal insufficiency, using a thrice daily regimen of 10 mg hydrocortisone. In the current study, a commercial immediate release 10 mg hydrocortisone tablet was used as a standard conventional hydrocortisone preparation.

The CPH pectin-based modified release formulations were prepared as multiparticulate encapsulated granules in gelatin capsules in view of their potential benefits in colonic drug delivery. Multiparticulate systems are associated with increased bioavailability, decreased systemic toxicity, decreased risk of dose dumping, reduced local irritation, and a potentially longer residence time in the ascending colon as compared to single unit systems [30].

Modulation of systemic absorption of drugs in the colon is an emerging approach in the treatment of diseases that display circadian variation [31, 32]. The performance and formulation characteristics of the modified release capsules were intended to delay drug release in the upper gastrointestinal tract after which drug release will be triggered by colonic bacteria [31]. The use of biodegradable polymers such as pectin in the design of modified release capsules holds great promise in targeted release of hydrocortisone in the colonic region.

The use of matrix systems in targeted delivery is associated with a rapid initial release of drug, which is a challenge when delayed drug release is desired. Pectin is a hydrophilic polymer with the potential for premature drug release due to its high solubility in the gastrointestinal tract. To overcome this drawback, the CPH pectin-based hydrocortisone capsules were chemically modified with calcium, zinc, and HPMC to reduce their aqueous solubility [33]. Calcium binding with pectin ensures reduced solubility, increased gel strength, and extensive hydration in the gastrointestinal tract while inclusion of HPMC strengthens the hybrid polymer matrix [28]. Cross-linking of CPH pectin with zinc ions ensures reduced aqueous solubility of pectin [26]. Earlier reports suggest that zinc is a better cross-linker than calcium for pectin [26, 34, 35]. However, the inclusion of HPMC in Formulation A appeared to enhance the gel strength of the calcium and pectin drug matrix, leading to Formulation A (containing calcium and HPMC) and Formulation B (containing zinc and starch) showing similar drug absorption profiles.

Biphasic release, the process whereby drug release is initially delayed followed by accelerated drug release at a later stage is useful for colon specific drug delivery [36, 37]. Biphasic release is well-documented in press coated and film coated drug delivery systems but is rarely observed with matrix systems [28]. The biphasic release of Formulations A and B is potentially beneficial in the chronodelivery of hydrocortisone which may be useful in clinical conditions influenced by diurnal rhythms. Colonic delivery of drugs through biphasic or sigmoidal release mechanisms is useful for the maintenance of sustained blood drug levels [38].

In the current study, the time for drug to peak (T_max_) was longer for the modified release formulations than the commercial immediate release formulation. This confirms the delayed and extended release profiles of Formulations A and B. These modified release capsules may be useful in achieving the cortisol overnight rise and morning peak in cortisol deficiency. This finding is similar to earlier clinical reports, where a novel formulation of modified release hydrocortisone formulation achieved a physiological nocturnal cortisol rise and morning peak [12]. In a related study, the administration of a 10 mg modified release hydrocortisone and 10 mg immediate release formulation resulted in a T_max_ of 7.83 h and 1.8 h for the modified release and immediate release, respectively [25]. Whitaker et al. also reported a mean T_max_ of 8.3 hours for DIURF 002, a modified release hydrocortisone formulation, whereas the immediate release hydrocortisone formulation had a T_max_ of 1 hour [10].

The maximum serum concentration (C_max_) was also higher for Formulations A and B than the commercial immediate release product, indicating a possible controlled absorption rate for the modified release formulations. An elimination half-life (t_1/2_) of 7.261 ± 3.12 h and 10.462 ± 4.425 h for Formulations A and B, respectively, demonstrates the prolonged effect of both modified release formulations compared with the immediate release hydrocortisone formulation. Furthermore, Formulation B, with the longest half-life, and C_max_ and T_max_, ideal for a delayed release hydrocortisone drug, could be a drug of choice if dosing per day needs to be reduced to improve patient compliance. The data obtained showed that the total drug exposure (AUC_0→∞_) was greater for Formulations A and B than the commercial formulation. The relative bioavailability (Rel. F) of the CPH pectin-based Formulations A and B was ~213% and 274%, respectively. This demonstrates the ability of the modified release hydrocortisone formulations to provide a greater extent of drug exposure with time in the experimental animals compared to the conventional dosage form [39]. Despite these results, the short sampling time used which led to an abrupt end of the* in vivo* pharmacokinetic profile of rats administered with Formulations A and B and the extrapolation of the postabsorption-elimination phases of respective concentration-time curves is deemed a limitation of this aspect of the study.

Both CPH pectin composites of calcium and HPMC and zinc formulations were robust enough to successfully delay the release of hydrocortisone in the upper gastrointestinal tract, increase the residence time of hydrocortisone in the plasma, and increase bioavailability. However, Formulation A exhibited a better control of drug release than Formulation B. In situ cross-linking of CPH pectin with calcium and zinc ions played a major role in retarding the release of hydrocortisone. Zinc pectinate composites have been associated with slower initial release of drugs from previous reports [35]. However, the extent of initial release of hydrocortisone from Formulation B can be attributed to the influence of starch. Earlier reports indicate the potential rapid release of drugs from starch based systems due to extensive swelling and enzymatic degradation in the gastrointestinal tract [40]. The results suggest possible accelerated release of cortisol from Formulations A and B on arrival in the colon, after microbial degradation of pectin [31].

The nocturnal burst release observed with the modified release formulations is critical for restoring the cortisol peak associated with the disruption of cortisol rhythm. The extended release profiles of CPH pectin-based formulations should aid in meeting levels of overnight rise and early morning cortisol peak required in adrenal insufficiency.

## 5. Conclusion

This* in vivo* investigation of CPH pectin-based hydrocortisone multiparticulate capsules has shown that cortisol can be released in a circadian fashion in Sprague-Dawley rats and the formulation holds promise in achieving the chronodelivery of hydrocortisone in adrenal insufficiency in humans. There is the need, however, for further clinical investigations in human subjects to confirm replicability.

## Figures and Tables

**Table 1:** Composition of CPH pectin-based hydrocortisone capsule formulations.

Capsule formulation	Hydrocortisone (mg)	CPH pectin (mg)	Calcium chloride (mg)	Zinc acetate (mg)	HPMC (mg)	Dicalcium phosphate (mg)	Starch (mg)
A	10	50	50	-	25	65	-
B	10	50	-	50	-	-	90

**Table 2:** Pharmacokinetic parameters of the two CPH pectin-based hydrocortisone capsule formulations compared with a commercial immediate release hydrocortisone tablet.

Pharmacokinetic parameters	Commercial hydrocortisone tablet	Formulation A	Formulation B
C_max_ (ng/ml)	15.32 ± 0.31	21.80 ± 1.99*∗*	20.84 ± 2.66*∗*
T_max_ (h)	1 ± 0.20	6 ± 0.23*∗∗*	6 ± 0.35*∗∗*
t_1/2_ (h)	1.92 ± 1.48	7.26 ± 3.12^**φ**^	10.46 ± 4.43^**φ**^
AUC_0→∞_(ng.hr/ml)	42.78 ± 4.207	91.147 ± 2.013^**φ**^	117.463 ± 4.845^**φ**^
Rel. F (%)	100	213^**φ**^	274^**φ**^

*∗*p<0.05; *∗∗*p<0.001

^**φ**^: results obtained after extrapolations from concentration-time curves.

## Data Availability

The* in vitro* and* in vivo* pharmacokinetics data used to support the findings of this study are included within the article and are also available from the corresponding author by request.
